# Increased Gibberellins and Light Levels Promotes Cell Wall Thickness and Enhance Lignin Deposition in Xylem Fibers

**DOI:** 10.3389/fpls.2018.01391

**Published:** 2018-09-20

**Authors:** Renan Falcioni, Thaise Moriwaki, Dyoni Matias de Oliveira, Giovana Castelani Andreotti, Luiz Antônio de Souza, Wanderley Dantas dos Santos, Carlos Moacir Bonato, Werner Camargos Antunes

**Affiliations:** ^1^Laboratório de Ecofisiologia Vegetal, Departamento de Biologia, Universidade Estadual de Maringá, Maringá, Brazil; ^2^Laboratório de Bioquímica de Plantas, Departamento de Bioquímica, Universidade Estadual de Maringá, Maringá, Brazil; ^3^Laboratório de Histotécnica e Anatomia Vegetal, Universidade Estadual de Maringá, Maringá, Brazil

**Keywords:** lignin monomers, stem, hormones, cambial activity, cell wall

## Abstract

Light intensity and hormones (gibberellins; GAs) alter plant growth and development. A fine regulation triggered by light and GAs induces changes in stem cell walls (CW). Cross-talk between light-stimulated and GAs-induced processes as well as the phenolic compounds metabolism leads to modifications in lignin formation and deposition on cell walls. How these factors (light and GAs) promote changes in lignin content and composition. In addition, structural changes were evaluated in the stem anatomy of tobacco plants. GA_3_ was sprayed onto the leaves and paclobutrazol (PAC), a GA biosynthesis inhibitor, via soil, at different irradiance levels. Fluorescence microscopy techniques were applied to detect lignin, and electron microscopy (SEM and TEM) was used to obtain details on cell wall structure. Furthermore, determination of total lignin and monomer contents were analyzed. Both light and GAs induces increased lignin content and CW thickening as well as greater number of fiber-like cells but not tracheary elements. The assays demonstrate that light exerts a role in lignification under GA_3_ supplementation. In addition, the existence of an exclusive response mechanism to light was detected, that GAs are not able to replace.

## Introduction

Changes in plant habitat luminosity are associated to high phenotypic plasticity (Valladares et al., 2000; Pearcy, 2007; Pugnaire and Valladares, 2007; Sarlikioti et al., 2011; Niinemets et al., 2015), especially in relation to stem development (Pearcy, 2007; Kurepin and Pharis, 2014) and either endogenous gibberellin (GAs) levels or the signaling cascade triggered by this hormone (Alabadí et al., 2008; Lau and Deng, 2010).

Plants grown in high irradiance environments present reduced stem length and shorter internodes compared to shade-grown plants (Pugnaire and Valladares, 2007; Lau and Deng, 2010; Ribeiro et al., 2012; Wang and Benning, 2012; Kurepin and Pharis, 2014; de Wit et al., 2016). They also present higher chlorophyll and carotenoid concentrations per unit of leaf area (Pearcy, 2007; Pugnaire and Valladares, 2007; Lambers et al., 2008), thicker leaves, more elongated palisade parenchyma cells (Lambers and Poorter, 2004; Poorter et al., 2009) and increased tissue lignification (Onoda et al., 2017), among other biochemical and anatomical alterations that provide acclimatization to high irradiance conditions (Givnish, 1988; Kurepin et al., 2007; Pugnaire and Valladares, 2007). On the other hand, plants grown in low irradiance environment display shade avoidance syndrome with etiolated phenotype and lower lignin concentrations per biomass unit (Hoffmann and Poorter, 2002; Kurepin et al., 2007; Pugnaire and Valladares, 2007; Lau and Deng, 2010; Kurepin and Pharis, 2014; Hedden and Sponsel, 2015).

Among the various plant hormones synthesized by plants, GAs belong to a group of tetracyclic diterpenoids that promote biochemical, physiological and anatomical plant changes (Biemelt et al., 2004; Yamaguchi, 2008; Dayan et al., 2012; Hedden and Thomas, 2016). Three major enzymes that regulate GAs homeostasis and biosynthesis are known, namely GA 20-oxidase (GA20ox) and GA 3-oxidase (GA3ox), which catalyze the early phases of bioactive GA biosynthesis, and GA 2-oxidase (GA2ox), involved in the deactivation of bioactive GAs (Yamaguchi, 2008; Martins, 2013; Hedden and Thomas, 2016). A fine metabolic regulation in response to light causes GA homeostasis to be controlled by induction of GA2ox genes, with negative feedback, as well as the GA20ox and GA3ox genes. GAs, in addition to several well-known responses (Ueguchi-Tanaka et al., 2007; Yamaguchi, 2008; Dayan et al., 2010; Aloni, 2013; Bai et al., 2014) also play a regulatory role concerning terpenes and phenolic compounds, particularly lignin (Hedden and Phillips, 2000; Olszewski et al., 2002; Biemelt et al., 2004; Davies, 2010; Ribeiro et al., 2012; Kurepin and Pharis, 2014; Ye et al., 2015; Hedden and Thomas, 2016). Together, light availability and GAs influence plant cell development and cell wall composition.

The changes observed at structural and ultrastructural stem levels is an end point of sequence of events that initiate with plant perception of surrounding environment, signaling amplifying, changes in gene expression and downstream proteomic/metabolic profiling modifications and concluding with cell developmental alterations. Changes in cell wall formation, structure, and composition (e.g., lignin deposition) are associated with intense cross-talk between light perception pathways (Onoda et al., 2017) triggered by photoreceptors, such as cryptochromes (Ahmad and Wani, 2014) and phytochromes (Casal, 2012). Furthermore, responses associated to hormonal signaling (Kurepin et al., 2007; Kurepin and Pharis, 2014), regulation of gene expression patterns, and cellular changes that result in metabolic changes (Yamaguchi, 2008; Ye et al., 2015; Hedden and Thomas, 2016; Zhang et al., 2018a), cytoskeleton structure (Bashline et al., 2014; Liu et al., 2016) and cell wall architecture (Zheng et al., 2017) are intertwined, creating a complex scenario of factors that regulate cell wall structure and composition.

Gibberellins also induce cellulose synthesis by promoting the release of secondary cell wall protein regulators (NAC SECONDARY WALL THICKENING PROMOTING FACTOR) (NST1–3) via DELLA repression cascade, which, in principle, could possibly boost and correlate with lignin deposition and increased lignin content (Zhao, 2016). Thus, it is suggested that a “normal” cellulose deposition pattern may be necessary for correct assembly and lignin deposition in primary, secondary and tertiary cell walls (Burk and Ye, 2002; Bai et al., 2014; Ye et al., 2015; Liu et al., 2016; Zhao, 2016; Zhang et al., 2018a).

Increases in GA contents stimulate lignin deposition under high irradiance conditions (Wada et al., 2005; Kurepin et al., 2006; Moura et al., 2010; Bai et al., 2014; Yang et al., 2015; Hedden and Thomas, 2016; Li et al., 2017; Zhang et al., 2018a). On the other hand, GA levels are higher under shaded conditions (Kurepin et al., 2006; Kurepin and Pharis, 2014), which in turn triggers cell stretching and etiolation, with lower lignin deposition over all secondary xylem (SX) fiber-like cells. Thus, lignification tends to be proportional to the amount of incident light, i.e., at lower luminous intensity, higher GA levels (Kurepin et al., 2006; Kurepin and Pharis, 2014) and lower lignin contents (and vice versa) are observed. This phenomenon indicates several knowledge gaps regarding individual light availability and GA responses to lignin deposition in plants, suggesting a cross-talk between light effects and GA effects on lignin metabolism. Above all, little is known on the interaction between GA levels and light on xylem fiber differentiation and lignin deposition on the cell wall (Dayan et al., 2012) or on the composition of lignin monomers (Wuddineh et al., 2015; Zheng et al., 2017) in response to light.

In this study, the hypothesis that light and GAs act independently in promoting tobacco plant structural xylem and cell wall modifications, lignin accumulation and stem monomer composition was tested. To do so, GA_3_ was supplemented via foliar spraying, and a GA biosynthesis inhibitor, paclobutrazol (PAC), an ent-kauren oxidase enzyme (KO; EC 1.14.13.78) was applied via soil (Ribeiro et al., 2012; Guo et al., 2015) in order to investigate cell wall, lignin and anatomical changes in tobacco plants under the influence of different GA levels in high and low irradiance environments.

## Materials and Methods

### Growth Conditions and Experimental Design

*Nicotiana tabacum* L. (cv. HAV 425) plants were cultivated in greenhouse conditions under full (100% sunlight) and low (low light – 8.5% of sunlight) irradiance levels. After initial growth in 5 L pots, one group was transferred to lower irradiance conditions inside the greenhouse, maintained by using two overlapping neutral nylon nets (80 and 50% shading) covering all plants until ground level reached 91.5% shading. No spectral quality differences were observed in this case, only decreased light intensity (data not shown). Gibberellic acid (GA_3_) at different concentrations was sprayed for 5 times every 2 days on leaves, while paclobutrazol (PAC) was applied to the soil. All plants grew for 20 days under these conditions. Reverse osmosis water was sprayed onto the controls (Cont), 10 μM GA_3_ (GA10), 100 μM GA_3_ (GA100) and PAC 50 mg L^−1^ (PAC), following the protocol reported by Falcioni et al. (2017), as well as in combined GA_3_ 10 μM + PAC (GA10P) and GA_3_ 100 μM + PAC (GA100P) treatments, totaling 12 treatments with 6 repetitions each. Representative plants are displayed in **Supplementary Figure S1**. The complete growth profile of these plants was evaluated following (Falcioni et al., 2018). Chloroplastidic pigments were quantified as reported by Lichtenthaler (1987).

### Epifluorescence Microscopy Analysis

Stem segments (∼2 cm^3^) were fixed in a modified Karnovsky solution containing 2.5% glutaraldehyde and 2% paraformaldehyde in 0.05 M phosphate buffer, pH 7.2 (Karnovsky, 1965) and stored at 4°C until processing. The stem samples were then washed in distilled water for 5 min, rehydrated and placed in glass containers. Subsequently, a 25% (w/v) aqueous polyethylene glycol 6000 (PEG 6000) solution was added to the samples and the material placed in an oven at 60°C. When half of the initial solution volume was reached, 75% (w/v), the PEG 6000 solution was again added. When the solution reached half the total volume again, the fragments were incorporated to a 90% PEG 6000 and gum arabic solution (Ferreira et al., 2017). The stem fragments were placed in cassettes mounted on a wood base with adhesive tape and immediately frozen at −16°C. Samples were then unformed, cut using a hand-rotated microtome (thickness 25–35 μm) and plated with water between 35 and 50°C (Souza et al., 2016; Ferreira et al., 2017). Staining was performed using astra blue and safranin/basic fuchsin 1% (w/v) (Kraus et al., 1998) and slides were then mounted between the slide and a cover slip with 50% glycerin. Digital images were obtained on an EKB-2F epifluorescence microscope (Eikonal Ind., São Paulo, Brazil) at the violet excitation wavelength (400 nm) (Ferreira et al., 2017). The images were processed with the Bel Eurisko software (Bel Photonics, Piracicaba, Brazil) and analyzed qualitatively and quantitatively for changes in vascular cylinder morphology with the Image-Pro-Plus^®^ v.4.5 software. All analyses were performed using cross sections of stem focusing on vascular cylinder in which composed by tracheary elements (tracheids and vessel elements), fibers (fiber-tracheids and libriform fibers) and parenchyma cells. In addition, some cell of primary xylem (ex. Protoxylem) were observed in slides. The term fiber-like cells refer to the all types of fiber cells on the axial system, with exception of the high differentiated vessel elements (Evert and Eichhorn, 2006).

### Fiber Microscopy Analysis

The acid maceration technique was employed for the quantitative fiber analysis, according to Johansen (1940) and Kraus and Arduin (1996). Briefly, the stem fragments fixed in Karnovsky’s solution (Karnovsky, 1965) were placed in 20 mL glass vials containing a 1:1 acidic solution containing 10% (v/v) chromic acid and 10% (v/v) nitric acid (Jeffrey’s solution) for 30 min and then washed in water (Johansen, 1940). An aliquot of the cell contents stained with safranin 1% (v/v) was arranged between the slide and the cover slip (Souza et al., 2016). The digital images were obtained on a microscope (Leica)^®^ coupled to a computer running the Leica Application Suite^®^ software. Measurements were performed using the Image-Pro-Plus^®^ v.4.5 software.

### Stem Scanning Electron Microscopy

For the vascular system basal region ultrastructure analysis of tobacco plant stems, approximately 1 cm^3^ stem segments obtained on the 20th treatment day were fixed in the previously mentioned modified Karnovsky’s solution (Karnovsky, 1965; Kiernan, 2000). The samples were subsequently infiltrated at different concentrations with a cryoprotectant (glycerol 10, 20, and 30%) until sinking. The fragments were then immersed in liquid nitrogen and fractured with a scalpel blade, the fragments were placed in a container with distilled water and dehydrated using an increasing series of acetone (30, 50, 70, and 90%) for 1 h and finally 3x (100%) for 10 min. Critical drying was carried out on a CPD-030 Bal-Tec critical point dryer (Bal-Tec AG, Balzers, Liechtenstein). The samples were assembled into stubs and metallized with gold on a MED010 Balzers evaporator (Bal-Tec AG, Balzers, Liechtenstein). Finally, the samples were observed on a SS-550 Shimadzu scanning electron microscope (Shimadzu, Tokyo, Japan) and the digital images were obtained with the SS-550 software^®^ coupled to the microscope and analyzed qualitatively.

### Transmission Electron Microscopy

For the transmission electron microscopy analysis, similar stem samples were fixed in modified Karnovsky’s solution (Karnovsky, 1965) with 2.5% glutaraldehyde and 2% paraformaldehyde in 0.05 M cacodylate buffer (pH 7.2) and post-fixed for 1 h with 1% osmium tetroxide in 0.05 M cacodylate buffer. The samples were then contrasted in bloc with 0.5% uranyl acetate overnight, dehydrated in an increasing acetone concentration series [30, 50, 70, 80, 90, and 100% (three times)], infiltrated and then polymerized into Spurr low viscosity epoxy resin. Sections (70 nm thick, Diamond Knife) were obtained using an ultramicrotome (MTX Powertome X, Boeckeler Instruments RMC Products) and contrasted with 3% uranyl acetate and lead citrate. Analyses were performed using a transmission electron microscope (JEOL JEM-1400, Leica Microsystems Inc., Buffalo Grove, IL, United States) equipped with a digital camera at 80 kV. All reagents were of high standard electron microscopy grade and purchased from Sigma (St. Louis, MO, United States) or EMS (Electron Microscopy Sciences, 1560 Industry Road Hatfield, PA, United States).

### Lignin Quantification and Monomer Composition

Tobacco dry stem powder (150 mg) was washed sequentially as follows: four times with 7 mL of 0.05 M potassium phosphate buffer pH 7.0, four times with 1% (v/v) Triton X-100 in 0.05 M potassium phosphate, three times with 1 M NaCl in phosphate buffer, twice with deionized water, and twice with acetone. After each washing step, the suspensions were centrifuged for 5 min at 1,400 × *g*. Finally, the pellets were dried at 60°C for 24 h and cooled in a vacuum desiccator. The obtained dry matter was defined as the protein-free cell wall (PFCW) fraction (Moreira-Vilar et al., 2014).

Total lignin content was determined using the acetyl bromide (AcBr) method (Moreira-Vilar et al., 2014). The PFCW fraction (20 mg) was placed in a screwcap centrifuge tube containing 0.5 mL of a freshly prepared acetyl bromide solution (25% v/v acetyl bromide/glacial acetic acid) and incubated at 70°C for 30 min. After complete digestion, the samples were quickly ice-cooled and mixed with 0.9 mL of 2 M NaOH, 0.1 mL 5 M hydroxylamine-HCl and 6 mL glacial acetic acid for complete solubilization of the lignin extracts. After centrifugation (1,400 × *g*, 5 min), the supernatant absorbance were measured at 280 nm. A standard curve was generated with alkali lignin (Aldrich 37, 095-9) and the results were expressed as mg lignin g^−1^ PFCW.

Lignin monomer composition was determined by alkaline nitrobenzene oxidation (Moreira-Vilar et al., 2014). This technique causes lignin degradation, forming *p*-hydroxybenzaldehyde from the H unit, vanillin from the G unit, and syringaldehyde from the S unit. PFCW aliquots (50 mg) was sealed in a Pyrex ampoule containing 1 mL of 2 M NaOH and 0.1 mL of nitrobenzene and heated to 170°C for 150 min, with occasional stirring. The samples were then cooled to room temperature, washed twice with chloroform, acidified to pH 3-4 with 5 M HCl and extracted twice with chloroform. The chloroform extracts were combined, dried, resuspended in 1 mL methanol and diluted in methanol/acetic acid 4% in water (20:80, v/v). Finally, the samples were filtered through a 0.45-μm disposable syringe filter and analyzed on a Shimadzu^®^ Liquid Chromatograph equipped with a LC-10AD pump, a CBM-101 Communications Bus Module, a Rheodyne^®^ injector and a SPD-10A UV-VIS detector. The compounds were separated on C18 column (150 mm × 4.6 mm, 5 μm; Supelco Discovery^®^ HS) with an equivalent pre-column (10 × 4.6 mm). The mobile phase comprised methanol/acetic acid 4% (20:80, v/v) at a flow rate of 1.2 mL min^−1^ in isocratic mode. Quantification of the monomer aldehyde products (*p*-hydroxybenzaldehyde, vanillin, and syringaldehyde) released by the nitrobenzene oxidation was performed at 290 nm using the corresponding standards. The results were expressed as mg monomer g^−1^ PFCW.

### Statistical Analyses

The quantitative data means were submitted to a One-Way ANOVA test for comparisons. Statistical significance was considered when *P* < 0.05 (Zar, 2010). Duncan’s test was applied to compare GAs levels in a particular light level (high or low light) and Student’s *t*-test was used to compare light levels in plants displaying similar GAs levels. In addition, Pearson’s correlation test was also performed when applicable. All statistical analyses were carried out using the STATISTICA 10^®^ software package (Statsoft, Tulsa, CA, United States). All graphs were prepared using the Sigma Plot 10.0^®^ (Systat, San Jose, CA, United States) software package.

A principal component analysis (PCA) (Jolliffe, 2002) was performed in order to reduce data dimensionality and to provide more statistically stable tests than other forms of regression for the set of autocorrelated variables. The PCA sought to evaluate the individual variable contributions in order to explain lignin contents under different light and GA regimes. The primary growth analysis data collected 20th days after treatment, as well as the data derived from the primary growth analysis and subsequent mathematical relationships were used to process the multivariate analysis. No vector rotation was performed. Only the first two main components were used for the other PCA-derived characterizations. Applying eigenvectors (linear correlation between a variable with a main component) and eigenvalues (square of the eigenvectors) allowed for statistical derivations concerning the magnitude of explanations of a particular variable (individually or groups) for accumulated lignin content and individual variable contribution. The score data estimated by the Principal Component 1 (PC1) and Principal Component 2 (PC2) were submitted to a bifactorial MANOVA and, if significant by the *F*-test (*P* < 0.05), were then submitted to a means comparison by Duncan’s test or the Student *t-*test, considered significant at an error probability of less than 5%.

## Results

In general, morphological changes were observed in the vascular cylinder, particularly in xylem fibers-like cells, with lignin content variations under the influence of direct GA_3_ and PAC applications, as well as in response to irradiance levels. Mitotic activity stimulation, with a greater number of xylem fibers-like cells in the cambial zone were found for both light and GA_3_ stimulation. As expected, GA_3_ supplementation induced increased internode length with similar number of leaves, except for PAC.

### Xylem Epifluorescence and Light Microscopy

**FIGURE 1:**
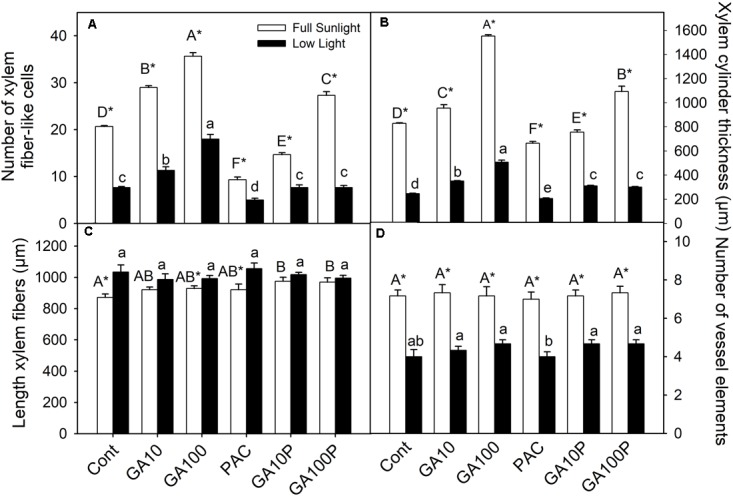
Number of xylem fiber-like cells counted in vascular cylinder radius **(A)**, xylem cylinder thickness **(B)**, length of xylem fibers **(C)** and number of vessel elements counted in vascular cylinder radius **(D)** of tobacco plants grown in high irradiance (full sunlight) and shade (8.5% of sunlight) environments and submitted to distinct gibberellin regimes. Cont (Control); GA10 (10 μM of gibberellic acid – GA_3_); GA100 (100 μM GA_3_) PAC (50 mg L^−1^ of paclobutrazol), and GA10P (combined GA_3_ 10 μM + PAC); GA100P (combined GA_3_ 100 μM + PAC). Different capital (full sunlight) or lowercase (shade) letters over bars indicate statistical difference between treatments on similar light regimes by Duncan’s test (*P* < 0.05). Asterisks indicate statistical difference between lights on similar GA regimes by Student *t* test (*P* < 0.05). (*n* = 6 ± SE).

**FIGURE 2:**
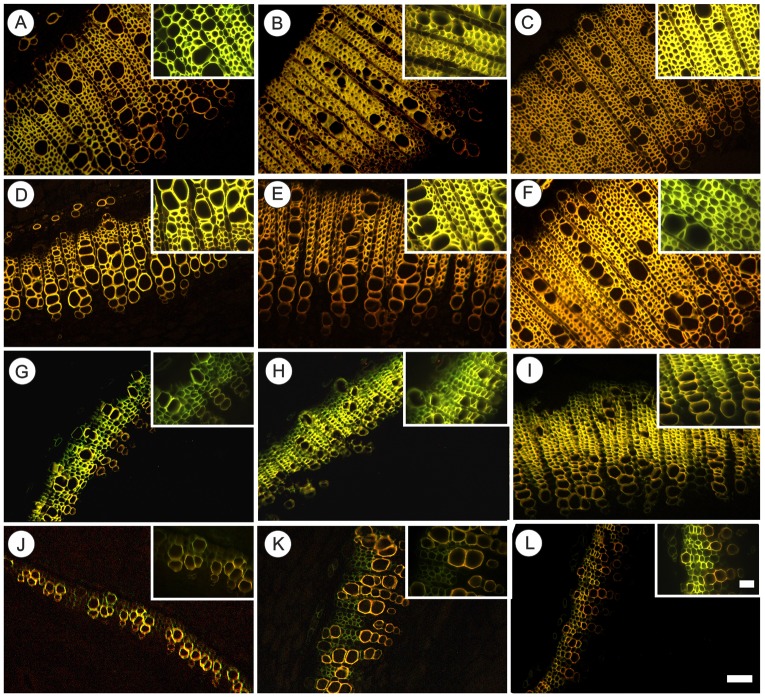
Fluorescence microscopy of cross section of basal region of representative stem of tobacco plants grown in high irradiance (**A–F**; full sunlight) and shade (**G–L**; 8.5% of sunlight) environments and submitted to distinct gibberellin regimes. Scale bar = 100 μm. Control **(A,G)**, GA10 **(B,H)**, GA100 **(C,I)**, PAC **(D,J)**, GA10P **(E,K)**, and GA100P **(F,L)**. Box sections for representative image in details fibers (Scale bar = 50 μm). For abbreviations of treatments, see **Figure 1**. Stained by safranin and astra blue in fluorescence microscopy under excitation violet light (400 nm).

The application of GA_3_, irrespectively of light levels, promoted an increase in the number of xylem fiber-like cells (>52%) (**Figures 1A,B**, **2**), higher cambial activity and the development of secondary wall structures (e.g., secondary xylem), whereas isolated PAC application promoted a strong repression (>37.5%) of their development, both in relation to the respective controls (**Figures 1A,B**, **2**). The number of vessel elements and fibers length were not altered (*P* > 0.05) between GA_3_ and/or PAC treatments (**Figures 1C,D** and **Supplementary Figure S3**).

GA_3_ application stimulated vascular cylinder thickening both in plants grown in full sunlight (>87%) (**Figures 1B**, **2C**) and in the shaded environment (>105%) (**Figures 1B**, **2I**), whereas plants treated with PAC only displayed decreased vascular cylinder thickness of 20.1% in the high irradiance environment (**Figures 1B**, **2D**) and 16.6% in the shade (**Figures 1B**, **2J**), both in relation to their respective controls (**Figures 2A,G**). In addition, plants treated with PAC and supplemented with GA_3_ (GA100P) presented phenotype recovering, with a vascular cylinder diameter increase of 31.9% in the sunlight-irradiated plants and 22.3% in the shaded plants (**Figures 1B**, **2F,L**), compared to their controls, whose increase was greater when compared to PAC.

### Scanning Electron Microscopy and Transmission Electron Microscopy

**FIGURE 3:**
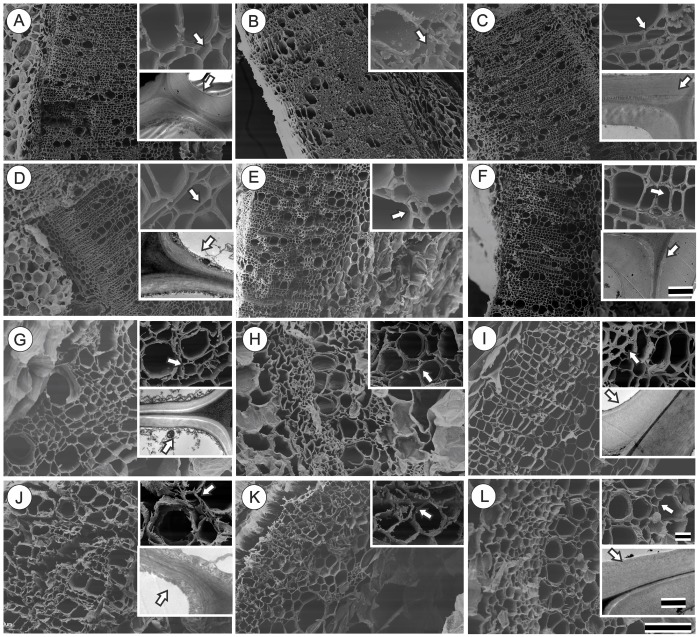
Scanning electron microscopy (SEM) and transmission electron microscopy (TEM) of transversal section of representative basal region of stem of tobacco plants grown in high irradiance (**A–F**; full sunlight; Scale bar = 500 μm) and shade (**G–L**; 8.5% of sunlight; Scale bar = 100 μm) environments and submitted to distinct gibberellin regimes Control **(A,G)**, GA10 **(B,H)**, GA100 **(C,I)**, PAC **(D,J)**, GA10P **(E,K)** and GA100P **(F,L)**. Box sections for representative image in details these cell wall thickness (Scale bar for SEM = 20 μm) and (Scale bar for TEM = 2 μm for full sunlight and 1 μm for low light). For abbreviations of treatments, see **Figure 1**. Arrows indicate details of cell wall of fiber-like cells.

An increase in cell wall thickness of xylem fiber-like cells in plants treated with GA_3_ (10 and 100 μM) independent of light conditions (**Figure 3**) was qualitatively verified. In addition, cell walls were well delimited when submitted to cryofracturing. In contrast, decreases in endogenous GAs levels through PAC application led to thinner and more fragile fiber walls in plants grown in both the full sunlight and the shade (**Figures 3D,J**) besides disuniformity electrodensity by TEM of cell walls. This phenotype was restored in the GA100P treatment (**Figures 3F,L**, inset). Regarding GA-treated plants supplemented with PAC, the cell wall of GA10P xylem fiber-like cells was partially restored (**Figure 3E**), while GA100P led to a complete restoration of fiber wall thickness (**Figure 3F**) in relation to the controls, in high irradiance conditions (**Figure 3A**). The ability of GA_3_ to reverse plant phenotype caused by PAC applications (**Figures 3K,L**) in shaded plants was observed in GA10P and GA100P treated plants, who presented thicker-walled fiber-like cells compared to plants treated with PAC only (**Figure 3J**), but thinner when compared to the GA10 and GA100 treatments (**Figures 3H,I**) in shade-grown plants. No evidence of changes in vessel elements wall thickness was detected in relation to GA_3_ and/or PAC treatments in any of the evaluated combinations herein in this work (**Figure 3**).

### Lignin Content and Monomer Composition

**FIGURE 4:**
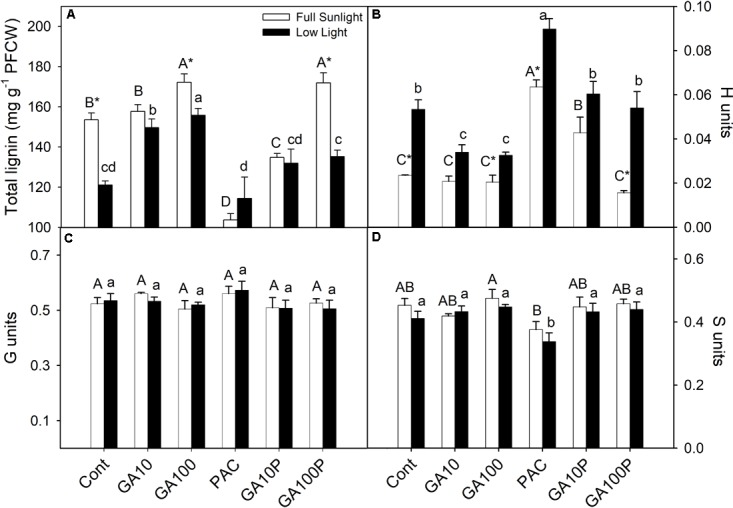
Lignin content in protein-free cell wall fraction **(A)**, monomer composition *p*-hydroxyfenil (H units) **(B)**, guayacyl (G units) **(C)**, syringyl (S units) **(D)**. For details of treatments and statistics, see **Figure 1**. (*n* = 3 ± SE).

The application of GA_3_ induced increased lignin contents in stems, of 12.4% for GA10 and 28.1% for GA100 plants in full sunlight conditions, whereas the PAC treatment led to a 32.6% decrease (**Figure 4A**) (*P* < 0.05). Similarly, in the shaded environment, lignin contents increased 23.1% in GA10 and 28.2% in GA100 plants, while PAC application decreased lignin contents in 5% (**Figure 4A**). GA_3_ application induced increased stem lignin contents in PAC-treated plants in both irradiance conditions (**Figure 4A**). Higher *p*-hydroxyphenyl (H) deposition was observed in shade plants (**Figure 4B**), although to a lesser extent compared to the syringyl (S) and guaiacil (G) monomers, which decreased in response to GA_3_ (**Figure 4B**). In addition, PAC-treated plants exhibited higher levels of the H monomer in both irradiance conditions (**Figure 4B**). No changes were detected (*P* > 0.05) in the amount of G or S monomers in response to light or GA_3_ (**Figures 4C,D**).

### Multivariate Analysis

**FIGURE 5:**
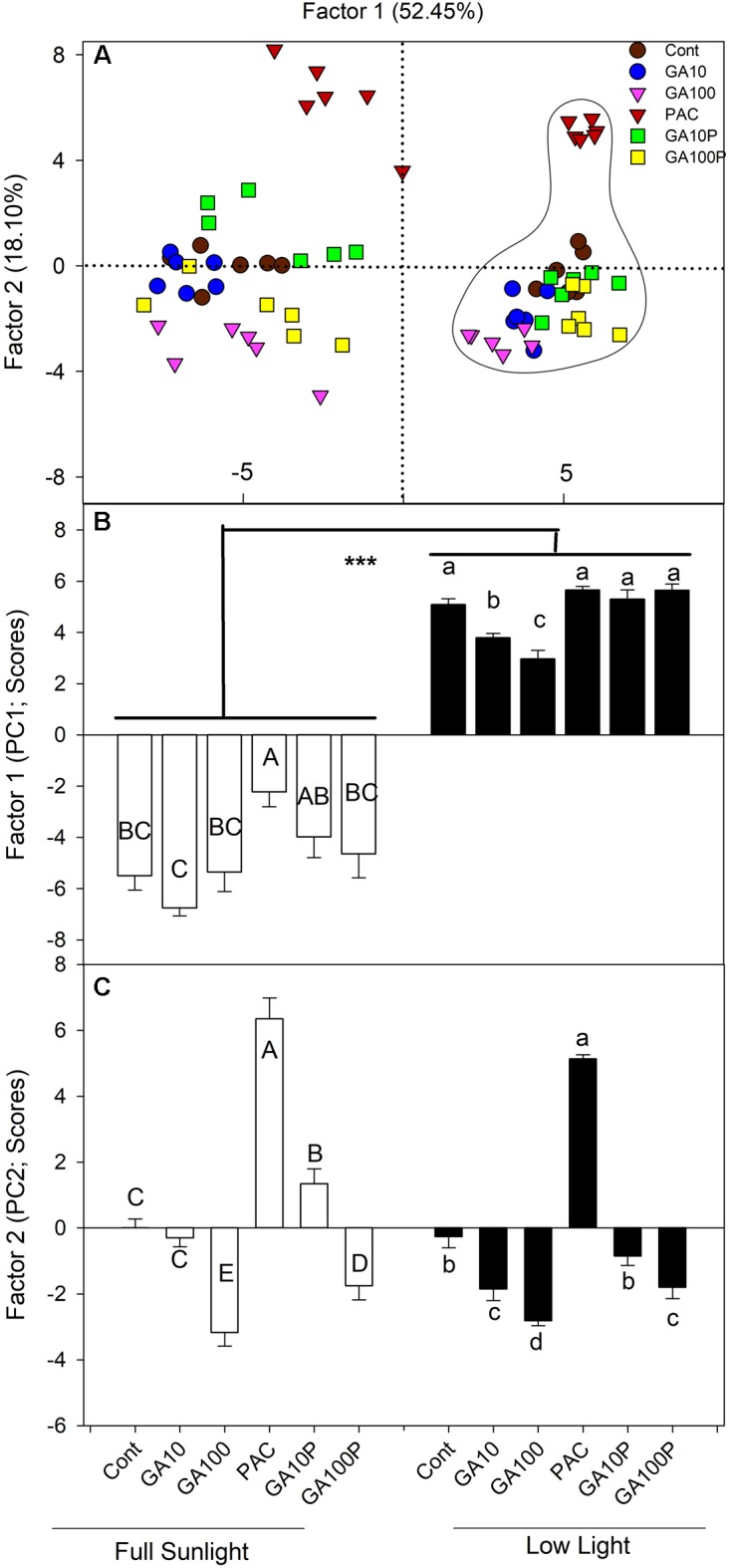
**(A)** Spatial distribution of the estimated scores of each replicate and treatment in relation to the Principal Component 1 (PC1) and the Principal Component 2 (PC2) and their respective percentages of the total variance explained by each of the components in relation to the lignin accumulation in stems. Dashed lines on the right grouping has been shaded plants. **(B,C)** Estimated scores of each replicate and treatment in relation to the Principal Component 1 (PC1) and the Principal Component 2 (PC2) and their respective percentages of the total variance explained by each of the components in relation to the lignin accumulation in stems. For details of treatments, see **Figure 1**. Different capital (full sunlight) or lowercase (shade) letters over bars indicate statistical difference between treatments on similar light regimes by Duncan’s test (*P* < 0.05). Asterisks indicate statistical difference between lights on similar GA regimes by Duncan’s test (*P* < 0.01). (*n* = 6 ± SE).

A PCA was carried out in order to estimate the individual contribution (or correlated groups) of the variables evaluated herein (raw data are not shown) regarding lignin accumulation in plant stems. The first component (PC1 – highest variance, 52.45%) was able to separate the applied treatments into two large groups (**Figure 5**). High participation of light levels was attributed to PC1 that, in isolation, allowed all for separation between high irradiance and low irradiance level conditions (**Figures 5A–C**).

The eigenvector analysis indicated that PC1 was strongly correlated with primary growth (root, stem, leaf and total plants dry matter and stem length) components, such as total DM (−0.9748), stem DM (−0.9326), stem diameter (−0.9178), energy cost (−0.9517), and total stem calorific energy (−0.9343) (**Supplementary Table S1**). In parallel, concerning PC2, the largest eigenvectors (in modulus) belonged to primary components and those derived from the growth analysis, such as internode length (−0.8449) and stem length (−0.6760), stem mass ratio (−0.8817) and number of fibers (−0.4514), identified as the individual variables most strongly associated to this component (**Supplementary Table S1**).

In order to further investigate the influence of the analyzed variables on lignin deposition, an empirical grouping of the different variables analyzed in six groups according to the “natural” experimental association of each group, namely primary growth, photosynthetic pigments, derived growth, calorific energy, biochemical and anatomical data, was performed. A high degree of correlation or autocorrelated variables (residuals are not independent) among the variables of each group was observed, making simple regression analyses inappropriate (Chatterjee and Hadi, 2012). For PC1, strongly associated to light, the highest explanation percentages were linked to the calorific group (25.8%), followed by the primary growth (19.8%) and anatomical (19.2%) groups, and, to a lesser extent, the biochemistry group (4.5%), concerning total data variability (**Supplementary Table S2**). However, for PC2, which was more strongly linked to GA levels, the highest percentage of explanation was related to the biochemistry group (32.8%), followed by the primary growth, pigments and derived growths groups (between 18.2 and 18.9%, respectively) concerning total data variability (**Supplementary Table S2**).

## Discussion

This study revealed an association between GA and light regarding the stimulation of lignin deposition in tobacco plant stems. GA_3_ partially (but not completely) replaced the effect of light on the stimulation of lignin deposition, particularly in xylem fibers. Our data indicate the existence of an exclusive response mechanism to light that GAs are not able to replace.

Lignin biosynthesis is affected in response to variations in light availability (Schopfer et al., 2001; Chen et al., 2002; Kimura et al., 2003; Syros et al., 2005; Andersson-Gunnerås et al., 2006; Yang et al., 2018). Unlike other reports, the experiments carried out herein demonstrate that light exerts a strong role in plant lignification under GA_3_ supplementation. In contrast, GAs are important in the stimulation of fibers lignification and differentiation, reducing the need for light during these processes. On the other hand, for treatments such as GA10P and GA100P in shaded conditions, supplementation with GAs was not enough to stimulate lignin deposition and fibers differentiation at levels similar to the respective counterparts grown in full sunlight (**Figures 2**, **3**, **4A**), indicating that light may be a necessary stimulatory component leading to high tissue lignification rates, when repressor mechanisms are acting or modulating lignin deposition. According to Kimura et al. (2003) and Roberts et al. (2008), many genes are expressed exclusively in high irradiance environments, so different mechanisms occur at high and low irradiance levels. Thus, light is a possible environmental components that stimulates the phenylpropanoid pathway (Roberts et al., 2008; Zhang et al., 2018a), as well as the expression of genes encoding important enzymes, such as PAL, CAD and PODs (Zhang et al., 2018b) which GAs alone are not able to replace.

Lignin biosynthesis does not only depend on the amount of the precursor components, but also on enzymatic activities, that are dependent on many other factors (e.g., gene expression, enzymatic regulation, interaction with phytochromes) (Zhao and Dixon, 2011; de Wit et al., 2016). Little is known so far at the transcription level (Wang et al., 2015b) and even less at the post-transcriptional level, such as how, and at what point (protein/metabolite), would the lignin biosynthesis pathway be affected primarily or mostly by GAs (Zhang et al., 2018a,b). In addition, it is not known whether some genes (and proteins) stimulated by GAs (Guo et al., 2015; Wang et al., 2015a; Li et al., 2017) suffer some kind of activity interaction and/or modulation by PIF proteins (Li et al., 2016). However, this study presents anatomical evidence triggered by this interaction. In *Arabidopsis thaliana*, genes correlated with lignin biosynthesis and associated to GAs variations are stimulated at high irradiance levels (Kimura et al., 2003; Moura et al., 2010). However, in general, wild plants grown in low irradiance exhibit higher GAs (Kurepin et al., 2006, 2007; Kurepin and Pharis, 2014) and lower lignin levels, according to the results reported herein (**Figure 4A**). That is, the less light, the lower the lignin content (and vice versa). By this direct analogy it would be possible to infer that the GAs act in an inhibitory (and not stimulatory) way in the lignin biosynthetic pathway, whereas light would play a fundamental role in the induction of this process. This study confirmed that both conditions stimulate the biosynthetic pathway linked to lignification, that lignin contents are not the direct reflection of the number of cells, and that GAs (GA_3_) promote fiber wall thickening and greater lignin content per cell, even in shaded environments, as evidenced by transmission electron microscopy (**Figure 3**, inset).

In the lignin biosynthetic pathway, a profuse metabolic flexibility and refined gene regulation is observed, as well as a complex interaction between these processes and the environment (Zhong and Ye, 2009; Zhao and Dixon, 2011). Linking data from other reports (Kurepin et al., 2007; Dayan et al., 2010; Kurepin and Pharis, 2014; Fukuda et al., 2016) with the present study, it is possible to speculate that, DELLA levels should be low at increased levels of GAs, as in plants grown in low-irradiance environments, since they are subject to degradation promoted by GAs (Ueguchi-Tanaka et al., 2007; Yamaguchi, 2008; Dixit, 2013; Racca et al., 2018), allowing PIF3 and PIF4 to be free to bind to their target genes. Shaded environments, although they lead to increased levels of endogenous GAs (Kurepin et al., 2006), appear to show poor interaction with PIFs, providing greater accumulation of DELLA proteins and inhibition of important enzymes, like PAL (Roberts et al., 2008; Locascio et al., 2013). GAs interact with the PIF (light dependent) complex, leading to the degradation of DELLA proteins and triggering the signaling required for growth induction (Li et al., 2016). In accordance to these results, in plants under the influence of PAC, which are expected to present lower endogenous GAs levels, light exerted a stimulatory role in the formation of fibers-like cells and lignin fiber wall deposition (**Figures 1B**, **2**, **3**). This combination is suggested to explain the decreased lignin contents observed in the stem over PAC under shade. Thus, while light controls PIF3 and PIF4 at the protein level (Soy et al., 2012; Zhong et al., 2012), GAs regulate their transcriptional activity (de Lucas et al., 2008; Li et al., 2016, 2017; Zhang et al., 2018a). This dual PIF3 and PIF4 regulation represents a point of integration (cross-talk) for the coordination of plant photomorphogenesis in response to light and GAs (Lau and Deng, 2010; Zhu et al., 2016). We emphasize that further enzymatic and gene expression studies in plants grown under controlled conditions undergoing light level manipulation should be performed to effectively confirm the data obtained by anatomical observations and direct quantification of lignin contents in stems (Guo et al., 2015; Zhu et al., 2016).

At the ultrastructural level, xylem fiber-like cells walls became thicker and more lignified as a result of increased GAs exposure, whereas decreased GAs through PAC applications lead to lower lignin contents in sun environments, but, especially, in shaded environments, making them fragile and less resistant (**Figure 3**), but not necessarily affecting stem diameter (Eriksson et al., 2000; Biemelt et al., 2004). It should be noted no evidence of changes in the number or thickening of vessel elements walls in response to GA levels was detected. In this sense, transcriptional factors of the MYB type, particularly MYB58, are stimulated, but not MYB63 (or *Arabidopsis thaliana* analogs) in triggering cell wall thickening via lignin deposition in the fibers but not in lignin increase in vessel elements (Zhou et al., 2009; Zhang et al., 2018b). However, there is no direct evidence to date that these transcriptional factors are stimulated by GAs or by light, only that they stimulate fiber-like cell wall and vessel elements thickening, specifically (Zhou et al., 2009).

### Multivariate Influence of Lignin Deposition

GA_3_ supplementation is able to stimulate lignification in shade-grown plants, but always at lower levels compared to plants in full sunlight (**Figure 4A**), indicating the existence of an exclusive response mechanism to light that GAs are not able to replace.

The multivariate analysis (PCA), that incorporated the effects of all the data collected, indicated that light was associated to the first component (PC1), which discriminated all high light treatments compared to low irradiance regards lignin content in stems. For the second component (PC2), cluster formation was observed. From the first to the last treatment (PAC, Control, GA10P, GA10 and GA100) supposedly with increasing GAs levels (**Supplementary Figure S1**). A growing response to increased GAs levels is observed, where the interaction between GAs and light appears to be additive in inducing cell lignification (**Figures 1–3**).

In full sunlight the percentage of explanation of lignin deposition were shared in many variables (**Supplementary Tables S1**, **S2**). In the shade, with limited C supply GA_3_ supplementation correlates strongly to plant height and internode length. GAs was capable of promoting phenotypic changes that trigger responses involved with biomass accumulation by maximizing light capture performance (leaf area density, **Supplementary Table S1**) in response to internode length (**Supplementary Figure S2A**) and greater efficiency of light interception by leaf area unit and number of leaves (**Supplementary Figure S2B**; Falcioni et al., 2018). In fact the increase in light interception also increases the photosynthetic rates, which would allow plants to accumulate greater DW, lignin and other components.

### GAs Effects on the Cell Wall Structure

Two experimental indications on the microfibrillar arrangement pattern were observed herein. First, more uniform uniformity of cell wall electrodensity (TEM) under GA_3_ action and the opposite under PAC applications (**Figure 3**, inset). This suggests a lack of uniformity in microfibril orientation under PAC applications and a reorientation with GA_3_ supplementation in PAC treated plants [as reported by Inada and Shimmen (2000) and Dixit (2013)]. Second, SEM analyses indicated that the fracture pattern was relatively smooth and uniform in full sunlight plants (except PAC-grown plants), while in the shade, particularly for PAC plants, a very irregular fragmentation pattern was observed (**Figure 3**).

A developed model concerning microtubule roles (Dixit, 2013) indicates that increased GAs availability may provide a larger microtubule organization and guide cellulose microfibrils in a transversal alignment, thus allowing for space filling with lignin (Inada and Shimmen, 2000; Dixit, 2013). Conversely, lower GAs concentrations direct cortical microtubule matrices to a greater cellular disorganization and a less dense network (Dixit, 2013), reducing the spaces to be filled by lignin. Appropriate organization provides spaces between microfibrils for lignin deposition (Li et al., 2016). The proposed model suggests that higher wall thickenings (and lignin deposition) would be the primary consequence of a simultaneous action promoted by light and GAs on microtubule accumulation and organization.

## Conclusion

The data presented herein reinforce the idea that light and GAs act exclusively in lignin promotion and deposition in tobacco plant stems, although a certain cross-talk between the routes is suggested. In high light availability environments, it is proposed that light and GAs must act in an additive way in promoting xylem fiber-like cellular differentiation. However, at low irradiance levels, even under high GA levels, lignin deposition is lower than the respective counterpart grown in high light availability, although a lignification stimulus also occurs, indicating a unique role played by light in these processes.

## Author Contributions

RF and WA designed the experiments, performed analysis, and wrote the manuscript. GA, CB, TM, DO, LS, and WS performed analyses and review the manuscript.

## Conflict of Interest Statement

The authors declare that the research was conducted in the absence of any commercial or financial relationships that could be construed as a potential conflict of interest.
