# Supplementary material for: Increased Gibberellins and Light Levels Promotes Cell Wall Thickness and Enhance Lignin Deposition in Xylem Fibers
Source: Front Plant Sci. 2018 Sep 20;9:1391. doi: 10.3389/fpls.2018.01391 (PMC6158321; doi:10.3389/fpls.2018.01391)
Supplement: Supplementary file 1 [file Table_1.DOCX]

Supplementary Material

Increased gibberellins and light levels promotes cell wall thickness and enhance lignin deposition in xylem fibers

Renan Falcioni^1,2*^, Thaise Moriwaki^1^, Dyoni Matias de Oliveira^2^, Giovana Castelani Andreotti^1^, Luiz Antonio de Souza^3^, Wanderley Dantas dos Santos^2^, Carlos Moacir Bonato^4^, Werner Camargos Antunes^1,2*^

*** Correspondence:** Corresponding Author: wcantunes@yahoo.com; wcantunes@uem.br and renanfalcioni@gmail.com

# Supplementary Figures and Tables

Figures: 03

Tables: 02

## Supplementary Figures


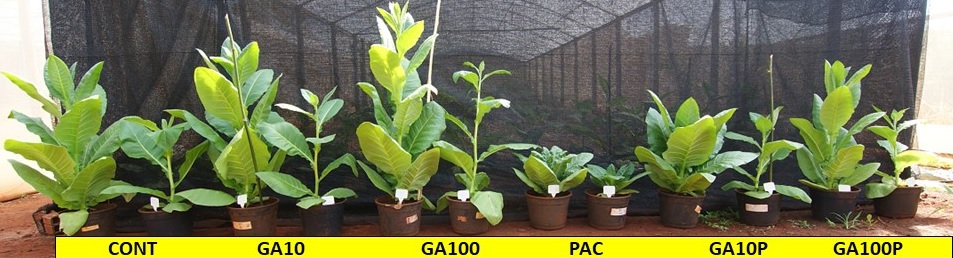


**Supplementary Figure 1.** Representative image of tobacco plants. Grown in high irradiance (full sunlight, left in each pair) and shade (8.5% of sunlight, right in each pair) environments and submitted to distinct gibberellin regimes. From left to right the treatments Cont (Control); GA10 (10 µM of gibberellic acid - GA_3_); GA100 (100 µM GA_3_) PAC (50 mg L^-1^ of paclobutrazol), and GA10P (combined GA_3_ 10 µM + PAC); GA100P (combined GA_3_ 100 µM + PAC).


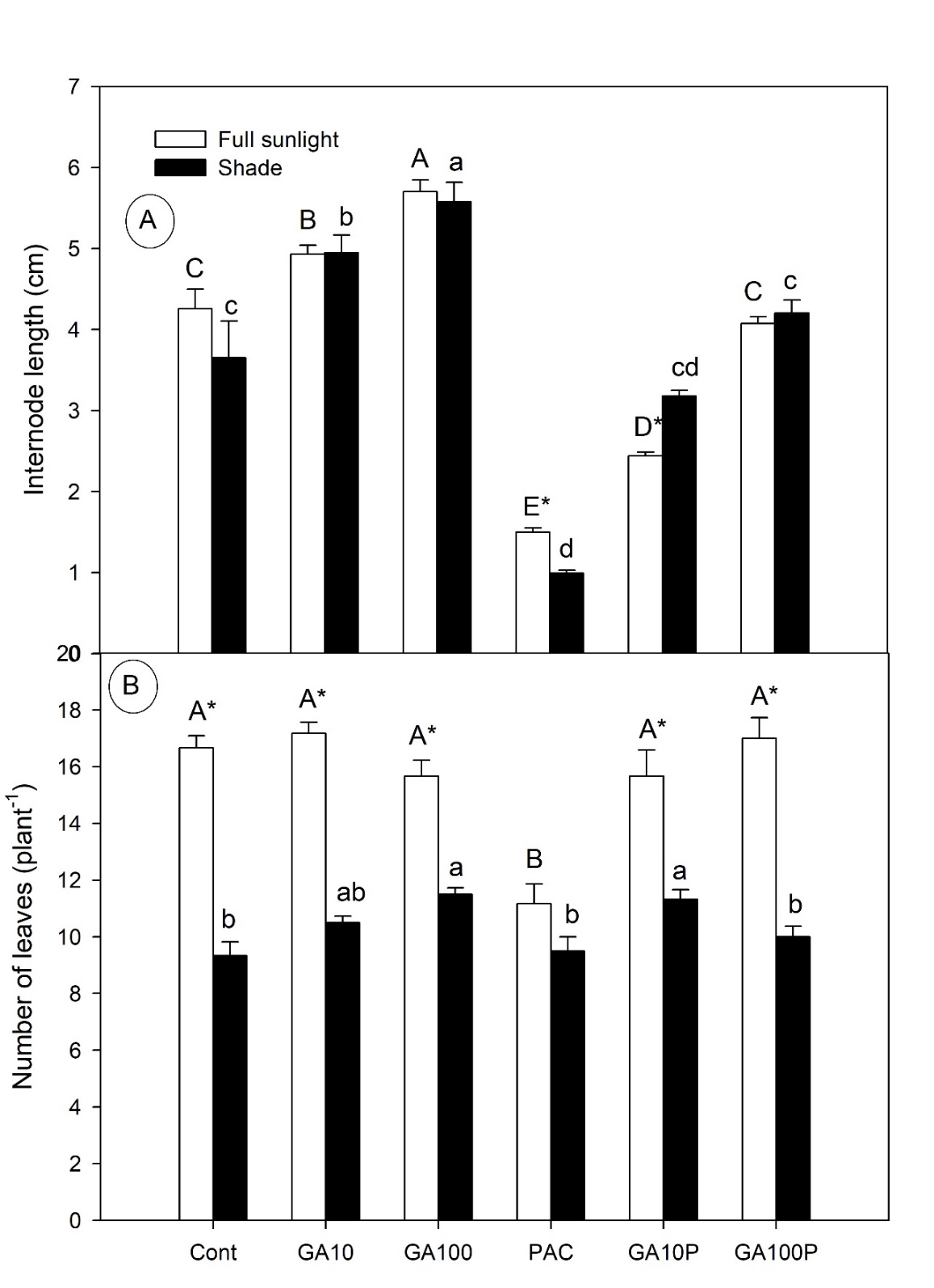


**Supplementary Figure 2**. (A) Mean of internode lengths and (B) number of leaves (> 5cm) of plants submitted to distinct gibberelins or light levels. Cont (Control); GA10 (10 µM of gibberellic acid - GA_3_); GA100 (100 µM GA_3_) PAC (50 mg L^-1^ of paclobutrazol), and GA10P (combined GA_3_ 10 µM + PAC); GA100P (combined GA_3_ 100 µM + PAC). Means followed by upper or lower case letters represent statistical differences by Duncan’s test (P <0.05) among different GA levels within the same irradiance level. Asterisks over the means represent statistical differences by Student’s *t* test (P <0.05) between different irradiance levels at the same GA regimen. n = 6 ± SE.


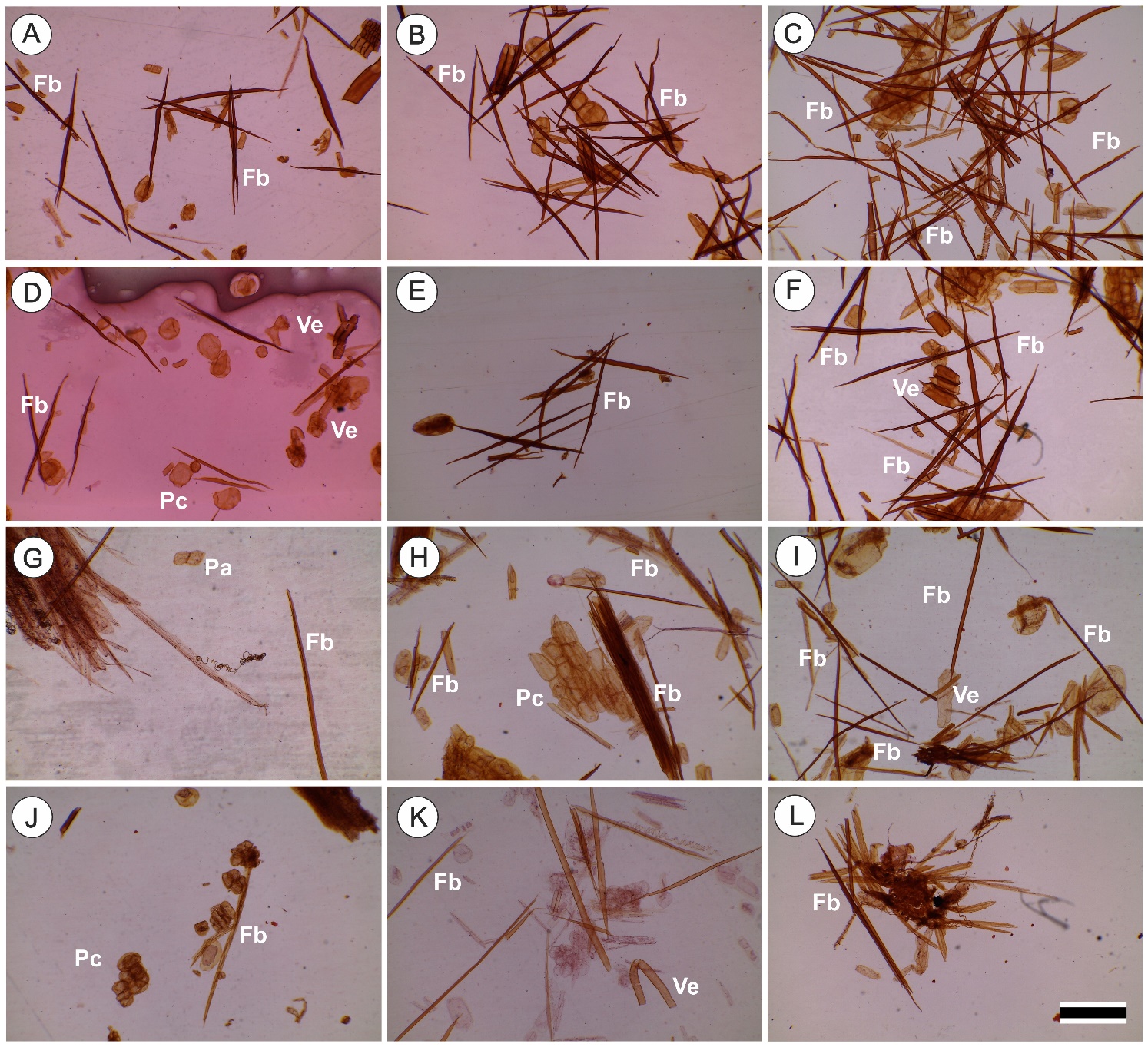


**Supplementary Figure 3.** Light microscopy of the macerated fibers of tobacco stems cultivated in high irradiance (full sunlight; A-F) and shade (8.5% sunlight; G-L) in response to variation of the content of GA3: Cont (A and G); GA10 (B and H); GA100 (C and I); PAC (D and J); GA10P (E and K) and GA100P (F and L) at 20 days. For abbreviations of treatments, see Figure 1 in main text. Dyeing with safranin 1%. Scale bar = 500μm. Ve = Vessel element; Fb = Xylem fibers; Pc = Parenchyma cells.

## Supplementary Table

**Supplementary Table S1**. Distribution of variables in relation to principal component (PC1) and the principal component 2 (PC2).

| Groups | Variables | Eigenvectors | | % of explication | |  |  |
| --- | --- | --- | --- | --- | --- | --- | --- |
|  |  | PC1 (*light*) | PC2 (*GAs*) | PC1 | PC2 | | |
| Growth (primary) | Root length (cm) | -0.4272 | 0.2539 | 0.710% | 0.727% | | |
|  | Root DW (g) | -0.8520 | 0.2983 | 2.824% | 1.003% | | |
|  | Stem length (cm) | -0.6428 | -0.6760 | 1.607% | 5.152% | | |
|  | Stem DW (g) | -0.9326 | -0.1794 | 3.384% | 0.363% | | |
|  | Stem diameter (cm) | -0.9178 | 0.1538 | 3.277% | 0.267% | | |
|  | Internode length (cm) | -0.2363 | -0.8449 | 0.217% | 8.047% | | |
|  | Leaf number | -0.8993 | -0.1898 | 3.146% | 0.406% | | |
|  | Leaf area (cm^2^) | -0.8960 | -0.1412 | 3.124% | 0.225% | | |
|  | Leaf DW (g) | -0.9714 | 0.0686 | 3.671% | 0.053% | | |
|  | Plant length (cm) | -0.6864 | -0.6101 | 1.833% | 4.197% | | |
|  | Plant DW (g) | -0.9748 | 0.0570 | 3.697% | 0.037% | | |
|  | Larger leaf (cm) | -0.7120 | -0.3856 | 1.972% | 1.676% | | |
|  | Cone volume (cm^3^ plant^-1^) | -0.7454 | -0.4484 | 2.162% | 2.267% | | |
|  | Leaf area density (cm^2^ cm^-3^) | 0.0141 | 0.8665 | 0.001% | 8.465% | | |
| Pigments | Chl *a* (mg m^-2^) | -0.1404 | 0.7119 | 0.077% | 5.714% | | |
|  | Chl *b* (mg m^-2^) | 0.1704 | 0.6960 | 0.113% | 5.461% | | |
|  | Chl total (mg m^-2^) | -0.0563 | 0.7437 | 0.012% | 6.236% | | |
|  | Carotenoids (mg m^-2^) | -0.3202 | 0.7149 | 0.399% | 5.763% | | |
|  | Chl *a*/Chl *b* (mg m^-2^) | -0.4889 | 0.0502 | 0.930% | 0.028% | | |
|  | Chl *a* (mg g^-1^) | 0.8704 | -0.0563 | 2.948% | 0.036% | | |
|  | Chl *b* (mg g^-1^) | 0.9074 | -0.0790 | 3.204% | 0.070% | | |
|  | Chl total (mg g^-1^) | 0.8969 | -0.0643 | 3.130% | 0.047% | | |
|  | Carotenoids (mg g^-1^) | 0.8902 | -0.1109 | 3.083% | 0.139% | | |
|  | Chl *a*/Chl *b* (mg g^-1^) | -0.4889 | 0.0502 | 0.930% | 0.028% | | |
| Growth derivates | Root mass fraction (g g^-1^) | -0.5499 | 0.5297 | 1.177% | 3.163% | | |
|  | Stem mass fraction (g g^-1^) | -0.1955 | -0.8817 | 0.149% | 8.764% | | |
|  | Leaf mass fraction (g g^-1^) | 0.6926 | 0.2990 | 1.866% | 1.008% | | |
|  | Leaf area fraction (cm^2^ g^-1^) | 0.9070 | -0.2444 | 3.201% | 0.674% | | |
|  | Specific leaf area (cm^2^ g^-1^) | 0.8365 | -0.3664 | 2.723% | 1.513% | | |
|  | A:R (g g^-1^) | 0.6846 | -0.3913 | 1.823% | 1.726% | | |
|  | LAI (cm^2^ cm^-2^) | -0.9017 | 0.1430 | 3.163% | 0.231% | | |
| Calorimetry | Root (cal g^-1^) | -0.7028 | 0.2228 | 1.922% | 0.560% | | |
|  | Stem (cal g^-1^) | -0.9517 | 0.1119 | 3.524% | 0.141% | | |
|  | Leaf (cal g^-1^) | -0.6826 | 0.0532 | 1.813% | 0.032% | | |
|  | DWT (cal g^-1^) | -0.8994 | 0.1533 | 3.147% | 0.265% | | |
|  | Root cost (kcal) | -0.8493 | 0.3082 | 2.806% | 1.071% | | |
|  | Stem cost (kcal) | -0.9343 | -0.1639 | 3.397% | 0.303% | | |
|  | Leaf cost (kcal) | -0.9738 | 0.0625 | 3.689% | 0.044% | | |
|  | DWT cost (kcal) | -0.9745 | 0.0691 | 3.695% | 0.054% | | |
|  | LA cost (cal cm^-2^) | -0.7962 | 0.4036 | 2.467% | 1.836% | | |
| Biochemistry | H - p-Hydroxybenzaldehyde | 0.6634 | 0.6192 | 1.712% | 4.323% | | |
|  | G - Guayacyl | -0.0497 | 0.4943 | 0.010% | 2.755% | | |
|  | S - Syringyl | -0.2632 | -0.7113 | 0.269% | 5.704% | | |
|  | S/G ratio | -0.1236 | -0.6063 | 0.059% | 4.145% | | |
| Anatomy | Leaf thickness (µm) | -0.6965 | 0.4376 | 1.887% | 2.158% | | |
|  | Secundary xylem thickness (µm) | -0.8514 | -0.2657 | 2.820% | 0.796% | | |
|  | Fiber xylem thickness (µm) | 0.5300 | 0.0228 | 1.093% | 0.006% | | |
|  | Vessel elements (number relative) | -0.8358 | 0.0472 | 2.718% | 0.025% | | |
|  | Fiber in vascular xylem (number relative) | -0.7882 | -0.4514 | 2.417% | 2.297% | | |
| **Total** |  | **-16.345** | **0.774** | **100%** | **100%** | | |

**Supplementary Table S2.** Explications of the estimated scores of each replicate and treatment in relation to the Principal Component 1 (PC1) and the Principal Component 2 (PC2) and their respective percentages of the total variance explained by each of the components in relation to the increased lignin accumulation of plants.

| **Groups** | **Factor 1 - *Light*** | **Factor 2 - *GAs*** |
| --- | --- | --- |
| Growth (primary) | 19.8% | 18.2% |
| Pigments | 13.0% | 18.2% |
| Growth derivates | 17.7% | 18.9% |
| Calorimetry | 25.8% | 3.7% |
| Biochemistry | 4.5% | 32.8% |
| Anatomy | 19.2% | 8.2% |
| **Total** | **100%** | **100%** |
